# An Intelligent Analysis Method for 3D Wheat Grain and Ventral Sulcus Traits Based on Structured Light Imaging

**DOI:** 10.3389/fpls.2022.840908

**Published:** 2022-04-13

**Authors:** Chenglong Huang, Zhijie Qin, Xiangdong Hua, Zhongfu Zhang, Wenli Xiao, Xiuying Liang, Peng Song, Wanneng Yang

**Affiliations:** ^1^College of Engineering, Huazhong Agricultural University, Wuhan, China; ^2^National Key Laboratory of Crop Genetic Improvement, National Center of Plant Gene Research, Huazhong Agricultural University, Wuhan, China

**Keywords:** wheat grain, ventral sulcus, structured light imaging, point cloud analysis, 3D traits

## Abstract

The wheat grain three-dimensional (3D) phenotypic characters are of great significance for final yield and variety breeding, and the ventral sulcus traits are the important factors to the wheat flour yield. The wheat grain trait measurements are necessary; however, the traditional measurement method is still manual, which is inefficient, subjective, and labor intensive; moreover, the ventral sulcus traits can only be obtained by destructive measurement. In this paper, an intelligent analysis method based on the structured light imaging has been proposed to extract the 3D wheat grain phenotypes and ventral sulcus traits. First, the 3D point cloud data of wheat grain were obtained by the structured light scanner, and then, the specified point cloud processing algorithms including single grain segmentation and ventral sulcus location have been designed; finally, 28 wheat grain 3D phenotypic characters and 4 ventral sulcus traits have been extracted. To evaluate the best experimental conditions, three-level orthogonal experiments, which include rotation angle, scanning angle, and stage color factors, were carried out on 125 grains of 5 wheat varieties, and the results demonstrated that optimum conditions of rotation angle, scanning angle, and stage color were 30°, 37°, black color individually. Additionally, the results also proved that the mean absolute percentage errors (MAPEs) of wheat grain length, width, thickness, and ventral sulcus depth were 1.83, 1.86, 2.19, and 4.81%. Moreover, the 500 wheat grains of five varieties were used to construct and validate the wheat grain weight model by 32 phenotypic traits, and the cross-validation results showed that the *R*^2^ of the models ranged from 0.77 to 0.83. Finally, the wheat grain phenotype extraction and grain weight prediction were integrated into the specialized software. Therefore, this method was demonstrated to be an efficient and effective way for wheat breeding research.

## Introduction

Wheat is one of the most important food crops in the world, which is the main food of approximately 40% global population (Hawkesford et al., 2013). With the rapid growth of the world population, wheat breeding and yield improvement are urgent to meet the growing demand for wheat (Reynolds et al., 2011; Fan et al., 2012; Ray et al., 2012; Lobos et al., 2017). It is well known that the weight of wheat grain would determine the final yield, and the grain shape characters that include grain length, width, thickness, volume are closely related to grain weight, which directly affects the yield and quality of wheat (Furbank and Tester, 2011; Reynolds et al., 2011). Increasing grain size and weight are the important research fields in wheat breeding (Tian, 2002). Meanwhile, the ventral sulcus, the unique morphological feature of wheat, is an important factor to the flour yield, which determines the wheat quality (Dziki and Laskowski, 2005; Le et al., 2019). Therefore, the measurements of wheat grain shape and ventral sulcus traits are significant and necessary to the wheat yield and quality-related research.

The traditional methods of grain measurement mainly rely on manual operation, which is inefficient, subjective, and labor intensive. With the increasing application of machine vision technology in agriculture, the method of phenotypic characters measurement based on image analysis has many advantages, such as high efficiency, high consistency, and non-destructive (Das Choudhury et al., 2019; Yang et al., 2020; Liang et al., 2021). At present, some open source software for image-based grain information measurement has been reported, such as SmartGrain (Tanabata et al., 2012), ImageJ (Schindelin et al., 2015), and WinSEEDLE (Ayele et al., 2017). In addition, Duan et al. (2015) developed a high-throughput automatic analysis system for rice yield-related traits, which includes total grain number, real grain number, 1,000 grain weight, grain length, and grain width. Komyshev et al. (2016) had realized the counting of wheat grains and the calculation of morphological traits on the mobile terminal. Huang and Chien (2017) developed image processing software based on the rice seeds RGB images to extract seven morphological features and classified three rice varieties. However, the above grain phenotype platforms all used three-dimensional (3D) imaging methods, which could only obtain the projected image information such as length, width, and area, while the grain thickness and volume were not able to be extracted. Moreover, the wheat ventral sulcus was complicated and difficult to measure, while the existing measuring method was based on the sliced measurement, which was destructive and unrepeatable, and it would be detrimental to the subsequent research. Therefore, it is urgent to develop a new method for wheat grain 3D and ventral sulcus traits measurement with non-destructive and high precision.

In the recent years, 3D imaging technology was developing rapidly, in which structured light imaging and X-ray computed tomography (CT) imaging technology were able to reconstruct the 3D structure with high precision. Structured light imaging was an active 3D vision technology and could obtain high-precision surface point clouds, which was widely used in industrial inspection, reverse engineering, and cultural relics protection (Paulus, 2019). CT imaging technology was originally used in the medical field, while it was also adopted in the non-destructive observation of small 3D structures such as roots and stems in crop phenotypes. Based on the 3D scanner, Yan and Long (2016) could obtain the corn seed boundary information with an automatic filling algorithm. Le et al. (2019) proposed a method to observe the wheat grains filling based on X-ray μCT imaging technique, while the ventral sulcus was also measured to describe the wheat grain development. Since the wheat grain size was distributed in 6–10 mm and the ventral sulcus was in sub-millimeter (Sun et al., 2007), the spatial resolution of 3D imaging should reach the micron level, and both the techniques could meet the demand (Strange et al., 2015; Liu and Wang, 2021). However, compared with CT 3D reconstruction, structured light imaging had the advantages of lower cost and high acquisition efficiency.

In this paper, an intelligent analysis method based on the structured light imaging had been proposed to extract the wheat grain 3D phenotypes and ventral sulcus traits. First, the 3D point cloud data of wheat grain were obtained by structured light scanner, and then, the specified point cloud processing algorithms that include single grain segmentation and ventral sulcus location have been designed. Finally, an integrated user software was developed for 32 wheat grain traits, which would contribute to the wheat grain-related research.

## Materials and Methods

### Materials

**FIGURE 1:**
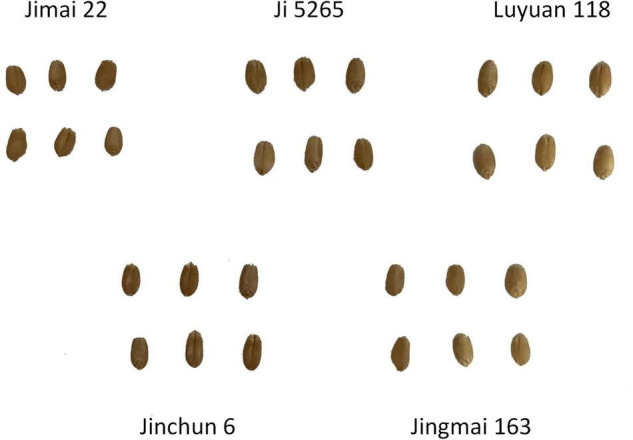
Experimental materials, including Jimai 22, Ji 5265, Luyuan 118, Jinchun 6, and Jingmai 163.

In this study, five commercial wheat varieties that have large differences in 1,000 grain weight were selected, which are generally distributed in 32–50 g. As shown in Figure 1, the varieties of wheat experimental materials include the following: Jimai 22, Ji 5265, Luyuan 118, Jinchun 6, and Jinmai 163. To evaluate the method accuracy, 25 seeds were randomly selected from each variety, and a total of 125 seeds were used for orthogonal experiment, while the comparison between structured light imaging and X-ray CT imaging was also conducted. In the prediction experiment of grain weight, 100 grains were selected from each wheat variety, and a total of 500 grains were used as the experimental materials.

### Experiment System

The 3D structured light scanner (Reeyee Pro, China) was adopted in the study, which was based on the white light LED raster scanning technology. Combing the advantages of structured light and binocular stereo vision, the scanner was able to achieve a single-sided accuracy of 0.05 mm within 2 s, which was suitable for high-precision scanning of small-sized work pieces. The equipment was mainly composed of a projector, two cameras, and an internal modulated light source. Based on the principle of triangulation and sinusoidal grating image, the dense point cloud data were able to be obtained. According to the different feature points of the scanned point clouds, the point clouds between various perspectives were merged to obtain the complete wheat grain. The scanner performance is shown in Table 1.

**TABLE 1:** Detailed parameters of Reeyee Pro scanner.

Parameter	Value
Light source	White LED
Point distance	0.16 mm
Spatial resolution	0.05 mm
Scanning area	210 × 150 mm
Working distance	290–480 mm
Maximum scan size	200 × 200 × 200 mm

### Experiment Design

#### Orthogonal Experiment

**FIGURE 2:**
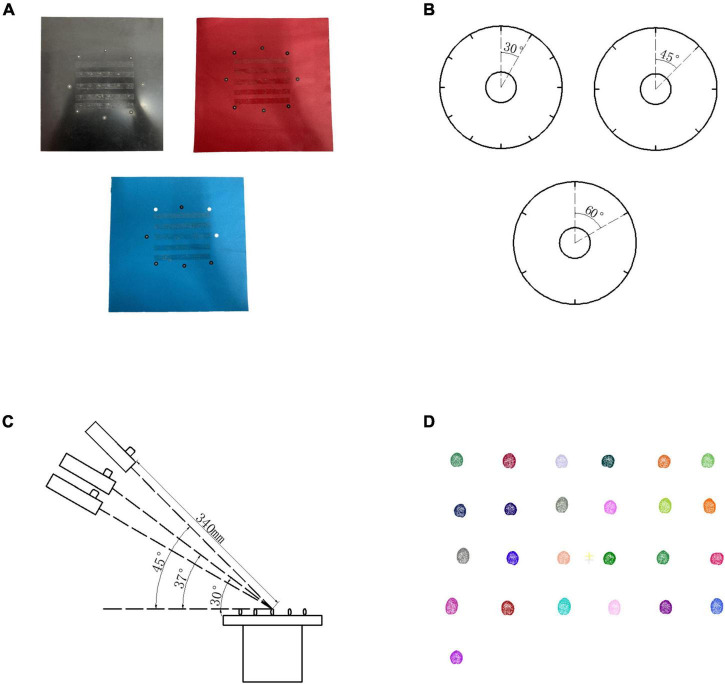
Orthogonal experiment design. **(A)** Wheat stages in 3 different colors. **(B)** Rotation method in different angle. **(C)** Scanning method in three different angles. **(D)** Wheat grain point clouds by structure light imaging.

Because Reeyee Pro scanner was based on the white light grating and triangulation principle, the background color of stage, scanning angle, and rotation angle of turntable would affect the reconstruction. To determine the best experimental conditions, 125 grains from 5 wheat varieties were used as the experimental materials. A total of three levels of orthogonal experiments (Huynh, 2011) were carried out on the three influencing factors that include background color, rotation angle, and scanning angle. The influence degree of each factor on the experiment was evaluated by four parameters: length, width, thickness, and ventral sulcus depth. As shown in Figure 2, the different levels of each factor are included (1) background color: black, red, and white; (2) rotation angle: 30°, 45°, and 60°; (3) scanning angle: 30°, 37°, and 45°.

#### X-Ray Computed Tomography Contrast Experiment

**FIGURE 3:**
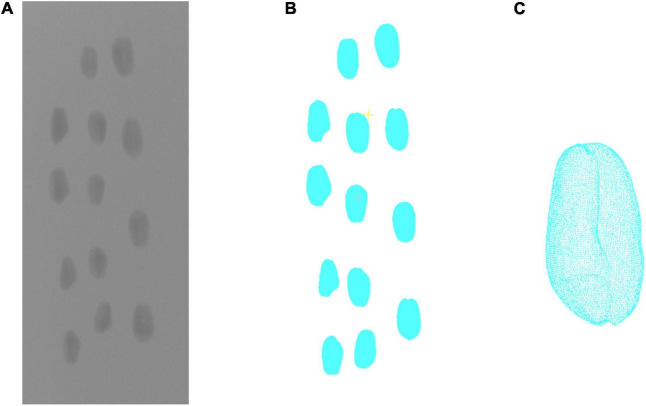
X-ray CT 3D reconstruction of wheat grains. **(A)** Projected image. **(B)** 3D reconstruction result. **(C)** Single grain point clouds.

To compare the result of structured light and X-ray CT imaging, the YXLON FF35 CT system was used in the contrast test. The system has adopted a water-cooled 220 kv output ray tube. Based on the spiral CT scanning mode, the pixel resolution of X-ray transmission image could reach 150 μm. First, the wheat grain was fixed in a foam stick. With the tube voltage of 60 kV and the tube current of 300 μA, 1,440 projection images were taken for X-ray CT rotation scanning of grains. Then, the projection image was used to reconstruct the wheat grain point cloud, which was shown in Figure 3. The projection image acquired by the X-ray detector had image size of 1,430 × 1,430 pixels and pixel size of 150 μm. According to the distance ratio of the X-ray tube to the detector and rotating table, the image magnification was about 5.94, whereas the spatial resolution was 25.23 μm per pixel. More performance parameters are shown in Supplementary Appendix 1. After the same point cloud processing scheme, the length, width, and thickness were calculated, whereas the X-ray CT measurement would be compared with the structured light measurement.

### Wheat Grain Point Cloud Processing Pipeline

**FIGURE 4:**
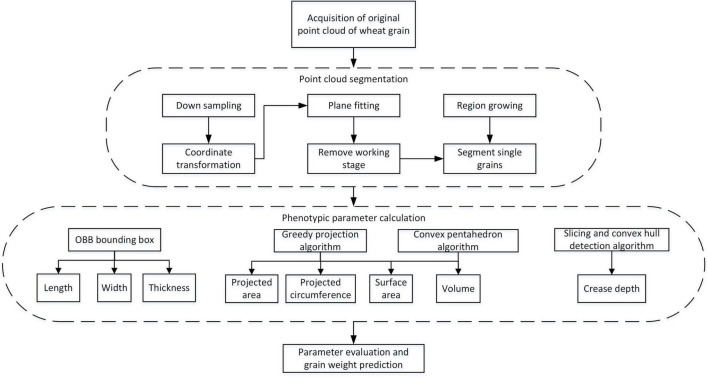
Wheat grain analysis pipeline.

After obtaining the original point clouds of wheat grain, the processing pipeline is depicted in Figure 4. It could be divided into three main steps: point cloud segmentation, phenotypic parameter calculation, and parameter evaluation and grain weight prediction, in which the point cloud processing algorithms that include downsampling, coordinate transformation, plane fitting, region growing, greedy projection, parameter extraction, and grain weight prediction were applied. In addition, the detailed processing procedures were described in the following.

**FIGURE 5:**
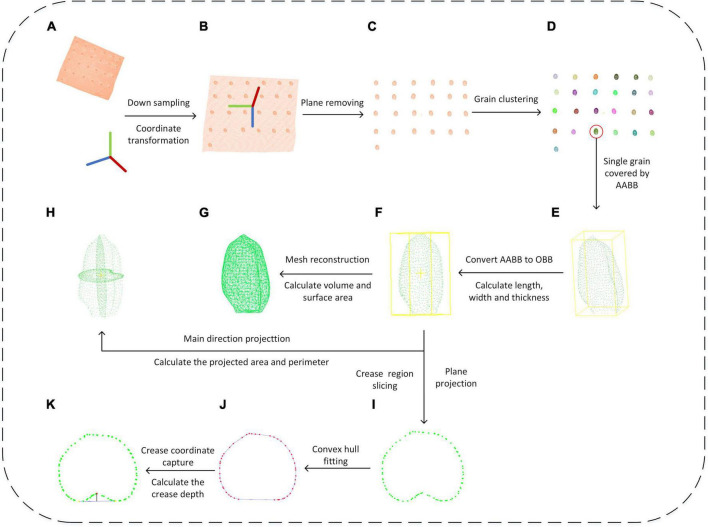
Wheat grain point cloud processing and trait extraction scheme. **(A–K)** Indicated the process of each step.

Since the dense point cloud was obtained by the structured light scanner (Figure 5A), to improve the computational efficiency of the subsequent algorithm, the voxel grid sample method was adopted (Chiang and Kuo, 2012). Meanwhile, to facilitate the subsequent object plane segmentation, the coordinate transformation of the initial point cloud was carried out, the process of which was shown in formula 1–3. First, the translation matrix *A* was computed by the centroid coordinate of the original point clouds, and then, the original wheat point cloud coordinate (*T*_0_) was moved to the centroid point with matrix *A*. After that, the covariance matrix (*M_T_*) was constructed based on the principal component analysis (PCA) (Wold et al., 1987), and the eigenvector was solved as the main direction of the wheat point cloud. Rotation and translation of the initial point cloud were carried out according to the centroid coordinates and the main direction of the original wheat point cloud to generate new coordinates. The converted result is shown in Figure 5B.


(1) $$T_{A}=M_{T}\times \left(T_{0}-A\right)$$

(2) $$A=\frac{\sum_{i=1}^{N}p_{i}}{N}$$

(3) $$M_{T}={\left\{e_{1},e_{2},e_{3}\right\}}^{T}$$

where, *P*_*i*_ is the i-th point coordinate of the original point clouds, and *N* is the number of points; *A* is the translation matrix; *T*_0_ is the original point cloud coordinates; *T_A_* is the new coordinates after coordinate transformation; Then, *e*_1_, *e*_2_, *e*_3_ are the three-unit eigenvectors of the covariance matrix*M*_*T*_.

After downsampling and coordinate conversion, the point clouds contained both grains and stage, which needed to be separated. The object stage point cloud could be regarded as a plane, which could be estimated by the plane fitting based on random sampling consistency (RANSAC) algorithm (Schnabel et al., 2007). Then, the original point clouds were segmented to obtain wheat grains point cloud, as shown in Figure 5C. With segmented grain point clouds, the discrete point removal was applied, and then, the multi grain point cloud was divided into single grain by region growth algorithm (Date et al., 2014). After that, the pseudo-colors were used to distinguish different wheat grains as shown in Figure 5D.

### Phenotypic Character Extraction

#### Length, Width, and Thickness Extraction

The extraction of length, width, and thickness was achieved by constructing OBB oriented bounding box from AABB Axis-aligned bounding box (Dimitrov et al., 2009). Based on the PCA algorithm, the segmented single grain point cloud (Figure 5E) was rotated and translated into consistent major axis direction (Figure 5F), and the new coordinate origin was at the geometric center of the grains. The maximum and minimum values of the transformed single grain point cloud in the new coordinate system were calculated as *x*_*max*_’*x*_*min*_’*y*_*max*_’*y*_*min*_’*z*_*max*_’*z*_*min*_, respectively. Finally, the grain length, width, and thickness were computed as following formula.


(4) $$l=x_{max}-x_{min}$$

(5) $$w=y_{max}-y_{min}$$

(6) $$h=z_{max}-z_{min}$$

where, *l*, *w*, and *h* are the length, width, and thickness of a grain, respectively.

#### Surface Area and Volume

With triangulation process, the grain surface was a triangular mesh composed of multiple triangular patches (Figure 5G). After the coordinates of the three vertices of the triangular patch were obtained, the area of each small triangle was calculated using Helen’s formula (Connelly, 2009) as Formula 7. The grain surface area was computed by the sum of all triangular patches areas as Formula 8. The 3D model of grain was a closed space surrounded by triangular mesh, and its volume V was calculated by the sum of the volumes of convex pentahedron projected by all triangular surfaces on the central plane, while the convex pentahedron volume was computed by the sum of three tetrahedral volumes (Zhang and Chen, 2001).


(7) $$s_{i}=\sqrt{p_{i}\left(p_{i}-a_{i}\right)\left(p_{i}-b_{i}\right)\left(p_{i}-c_{i}\right)}$$

(8) $$S_{a}=\sum_{i=1}^{k}s_{i}$$

where, *S_a_* is surface area of a grain, k is total number of triangles, *s_i_* is area of the i-th triangle, *p_i_* is half the perimeter of the triangle, and a_*i*_, *b*_*i*_, and *c*_*i*_ represent the length of each side of the triangle.

#### Projected Area and Perimeter Extraction

In this study, the grain point clouds were projected along three axis directions (Figure 5H), and the projected area and perimeter were obtained as the shape description of grain. The single grain point cloud after coordinate transformation was projected along X-, Y-, and Z-axes direction, respectively. Then, the projection point cloud was reconstructed and the area was calculated based on the greedy projection algorithm (Marton et al., 2009). The projected point clouds consisted of several triangular patches, while the boundary edges were identified by the edge sharing in the triangular patches. Finally, the perimeter was calculated by the sum of boundary edges.

#### Ventral Sulcus Traits

The measurement of grain ventral groove depth was performed according to the manual method. First, the wheat grain point cloud was intercepted based on through filtering of *X*-axis direction, the interception interval was set as 0.4 mm, and total of nine slices were extracted, while the central slice was at grain center. The slice point cloud was projected along the *X*-axis (Figure 5I), and the slice point cloud with the largest convex hull area was selected. The two furthest points in the convex hull were taken as the ventral sulcus edge points shown in Figure 5J. Based on RANSAC circle fitting, the nearest point to the circle center was regarded as the deepest point in ventral sulcus (Figure 5K). Finally, the depth of the ventral sulcus was calculated based on the distance from ventral sulcus deepest point to the line of ventral sulcus edge point. Meanwhile, the ventral sulcus slice point should be reordered based on the polar coordinate angle, and the Euclidean distance of each adjacent point was calculated and summed as the perimeter of the slice. Based on the Helen’s theorem (Connelly, 2009), the area of the triangle formed by the center point of the slice and each adjacent two points was calculated and summed as the area of the ventral sulcus slice. Additionally, the convex hull area subtracted the slice area as the ventral sulcus area.

### Parameter Evaluation and Grain Weight Prediction

A total of 32 parameters were extracted to quantitatively describe the wheat grains shape, which were consisted of 16 basic parameters and 16 derived parameters (Table 2). Roundness and sphericity (Shouche et al., 2001) were the two important shape parameters, which were used to characterize the grain shape in 2D and 3D. They were calculated by the formulas 9 and 10. mean absolute percentage error (MAPE), root mean square error (RMSE), and R^2^ were taken as the evaluation criteria in the system accuracy. They were computed as the formulas 11–13. After 500 wheat grains phenotypic datasets were obtained, five different machine learning algorithms, which include multivariable linear regression model, Bayesian ridge model, KNN regression model, random forest regression model, and gradient boosting regression model, were adopted to evaluate the wheat grain weight prediction model.


(9) $$c=\frac{4\pi S_{0}}{C^{2}}$$

(10) $$E=\frac{S_{e}}{S_{a}}$$

(11) $$MAPE=\frac{1}{n}\sum_{i}\frac{\left|x_{i}-y_{i}\right|}{x_{i}}$$

(12) $$RMSE=\sqrt{\frac{\sum_{i}{\left(x_{i}-y_{i}\right)}^{2}}{n}}$$

(13) $$R^{2}=1-\frac{\sum_{i}{\left(x_{i}-y_{i}\right)}^{2}}{\sum_{i}{\left(x_{i}-\bar{y}\right)}^{2}}$$

where, *c* is the roundness index; *C* is perimeter of cross-section; *S*_0_ is area of cross-section; *E* is sphericity; *S_e_* is a sphere surface area of equal the wheat volume; *S_a_* is the wheat surface area, *n* is the total number of measurements; *x_i_* is the manual measurement results; *y_i_* is the system measurement results; and $\bar{y}$ is the mean of the system measurements.

**TABLE 2:** The extracted 32 phenotypic traits of wheat grain.

No.	Symbol	Trait	No.	Symbol	Trait
1	*l*	Length	17	*l*/*h*	Ratio of Length to thickness
2	*w*	Width	18	*w*/*h*	Ratio of width to thickness
3	*h*	Thickness	19	*D*/*h*	Ratio of ventral sulcus depth to thickness
4	*V*	Volume	20	*V*_*obb*_	Box volume
5	*S*	Surface area	21	*S*/*V*	Specific surface area
6	D	Ventral sulcus depth	22	*S*/*l*	Ratio of surface area to length
7	*C*_*yz*_	Perimeter of cross section	23	*S*/*w*	Ratio of surface area to width
8	*S*_*yz*_	Area of cross section	24	*S*/*h*	Ratio of surface area to thickness
9	*C*_*xz*_	Perimeter of longitudinal section	25	*V*/*l*	Ratio of volume to length
10	*S*_*xz*_	Area of longitudinal section	26	*V*/*w*	Ratio of volume to width
11	*C*_*xy*_	Perimeter of horizontal section	27	*V*/*h*	Ratio of volume to thickness
12	*S*_*xy*_	Area of horizontal section	28	*S*_*s*_/*S*_*c*_	Ratio of ventral sulcus area to slice area
13	*C_c_*	Perimeter of slice	29	*c*_*yz*_	Roundness index of cross section
14	*S_c_*	Area of slice	30	*c*_*xz*_	Roundness index of longitudinal section
15	*S_s_*	Area of ventral sulcus	31	*c*_*xy*_	Roundness index of horizontal section
16	*l*/*w*	Ventral sulcus length to width ratio	32	E	Sphericity

### Software Design

The core configuration of the workstation used in this study was inter core i5 8300h, gtx1060, and the operating system was Windows 10. The development environment of point cloud processing algorithm was Microsoft Visual Studio 2015, open source C++ point cloud library (PCL), and visualization tool library visualization toolkit (VTK). The commercial software was used including Reeyee-Pro_v2.6.1.0, which was attached to the scanner and displayed the wheat grains point clouds, and VG studio 3.4 for X-ray CT 3D reconstruction.

**FIGURE 6:**
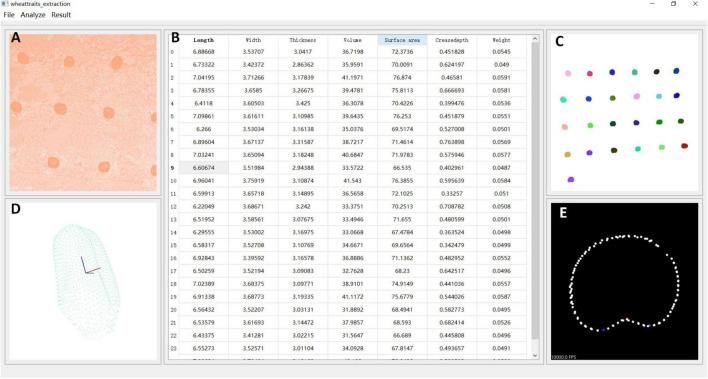
Wheat 3D traits extraction software. **(A)** Original point clouds. **(B)** Grain traits and weight prediction. **(C)** Grain segmentation results. **(D)** Single grain point clouds. **(E)** Ventral sulcus slicing results.

The wheat 3D traits extraction software is shown in Figure 6, and the interface was developed based on QT 5.9.8 and python 3.7, while the wheat grain point cloud processing algorithms that include point cloud segmentation, the wheat ventral sulcus extraction, grain traits calculation, and grain weight prediction were integrated. After setting the range of clustering points, the segmentation results of single grain would be extracted from the original point clouds, and then, wheat phenotypic characters would be calculated and displayed in the table. Meanwhile, the corresponding grain point cloud and ventral sulcus slice could be exhibited, when the data cell in the table was clicked. Finally, the wheat grain weight was predicted and displayed in the table. The detailed operation procedure of wheat 3D traits extraction software is shown in Supplementary Video 1. Additionally, the download address of the wheat 3D traits extraction software was provided freely for non-commercial research purposes^1^.

## Results

### Orthogonal Experiment Results

To evaluate each condition in the orthogonal experiment, the length, width, and thickness of 125 wheat grains were measured by three experimenters with vernier caliper, and the, average value was taken as the ground truth. After the measurement, the grains were sliced along *X*-axis to obtain the maximum cross-section, the top view image of which was acquired by backlight imaging. Then, the ventral sulcus point and two slice edge points were selected manually on the image, and the, ventral sulcus depth was calculated as the ground truth. The MAPE of length, width, thickness, and ventral sulcus was taken as the evaluation of orthogonal experiment. The results of the orthogonal experiment are shown in Table 3, and the original data of orthogonal experiment are shown in Supplementary Table 1, which indicated that the system had the best performance in the condition of the black object stage, 30° rotation angle, and 37° scanning angle.

**TABLE 3:** Orthogonal experimental results based on L9 (3^4) orthogonal table.

Test number	Factors			
	Stage color	Rotation angle	Scanning angle	Result
	
	Level			
	1	2	3	MAPE
1	1 (black)	1 (60°)	1 (30°)	0.0407
2	1 (black)	2 (45°)	2 (45°)	0.0551
3	1 (black)	3 (30°)	3 (37°)	0.0269
4	2 (red)	1 (60°)	3 (37°)	0.0901
5	2 (red)	2 (45°)	1 (30°)	0.0811
6	2 (red)	3 (30°)	2 (45°)	0.1048
7	3 (blue)	1 (60°)	2 (45°)	0.1826
8	3 (blue)	2 (45°)	3 (37°)	0.0932
9	3 (blue)	3 (30°)	1 (30°)	0.0913
K1	0.0409	0.1045	0.0710	
K2	0.0920	0.0764	0.1142	
K3	0.1224	0.0731	0.0701	
R	0.0815	0.0314	0.0441	

*Ki is the sum of the MAPE of the i level in different factors, R is the range of K.*

### Accuracy Analysis for Length, Width, Thickness, and Ventral Sulcus Traits

**FIGURE 7:**
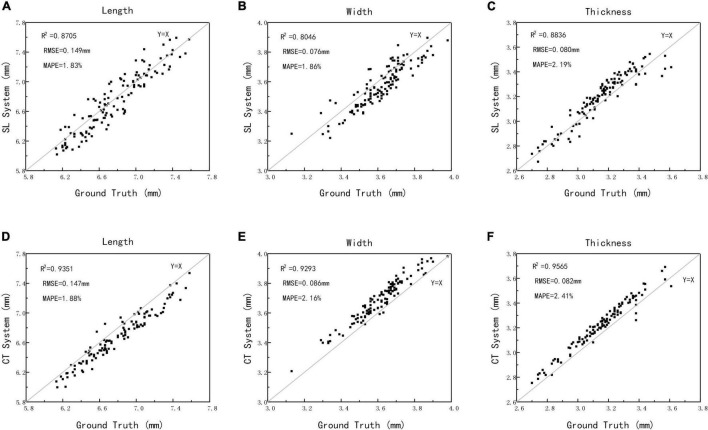
The automatic measurements vs. manual measurements of length, width, and thickness using structured light imaging **(A–C)** and X-ray CT imaging **(D–F)**.

To evaluate the structured light imaging performance, 125 wheat samples were compared with X-ray CT imaging results, in which the grain traits that include length, width, and thickness were both extracted. The measurement results are shown in Figure 7. The scatter plots of structured light measurement and ground truth are shown in Figures 7A–C, which showed that the R^2^, RMSE, and MAPE for length measurement was 0.8705 and 0.149 mm, and 1.83%, for the width measurement was 0.8046 and 0.076 mm, and 1.86%, and for the thickness measurement was 0.8836 and 0.080 mm, and 2.19%, respectively. Meanwhile, the scatter plots of X-ray CT measurement and ground truth are shown in Figures 7D–F, which showed that the R^2^, RMSE, and MAPE for length measurement was 0.9351 and 0.147 mm, and 1.88%, for the width measurement was 0.9293 and 0.086 mm, and 2.16%, and for the thickness measurement was 0.9565 and 0.082 mm, and 2.41%, respectively. The results demonstrated that the structure light measurement had high consistence with the manual measurement and was able to achieve the similar accuracy compared with X-ray CT measurements. Moreover, without X-ray radiation, the structured light imaging would be more effective in wheat ventral sulcus measurement.

**FIGURE 8:**
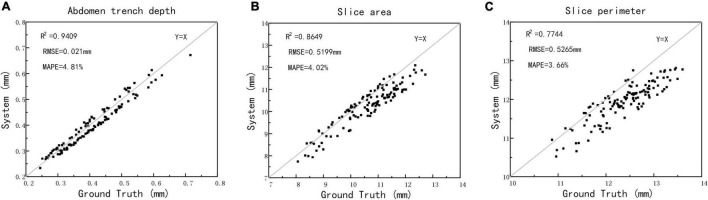
The automatic measurements versus manual measurements of wheat ventral sulcus traits. **(A)** Ventral sulcus depth. **(B)** Slice area. **(C)** Slice perimeter.

The method of manual cutting was used to obtain wheat grain slices, and then, the wheat slice image was taken by the visible light camera, while the depth, slice area, and slice perimeter of the ventral sulcus slice were extracted as ground truth. The scatter plots between structured light measurement and ground truth are depicted in Figure 8. The results showed that the R^2^, RMSE, and MAPE for depth measurement was 0.9409 and 0.021 mm, and 4.81%, for the slice area measurement was 0.8649 and 0.5199 mm, and 4.02%, and for the slice perimeter measurement was 0.7744 and 0.5265 mm, and 3.66%, respectively. The results demonstrated that this study could provide a non-destructive method for wheat ventral sulcus traits extraction with high accuracy. The original data of structured light imaging, X-ray CT, and manual measurements of 125 wheat grains are shown in Supplementary Table 2.

### Statistical Analysis and Grain Weight Prediction

**FIGURE 9:**
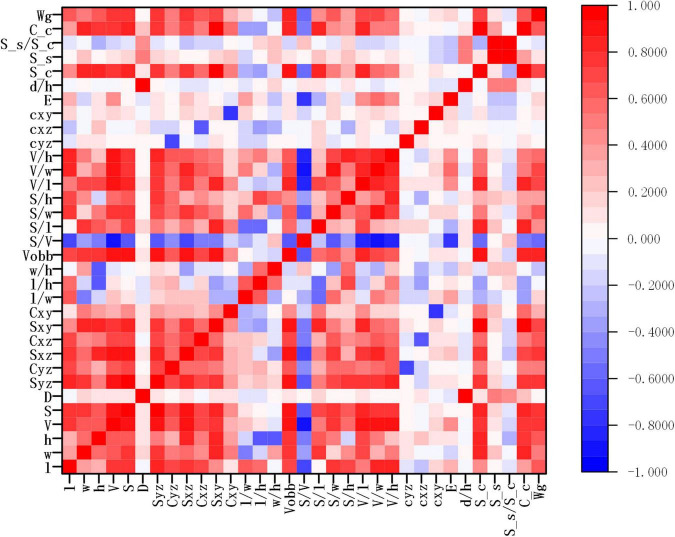
The result of wheat grain traits correlation analysis.

A total of 32 wheat grain traits were extracted in the study, while the correlation matrix of Pearson’s coefficient among parameters was calculated, and the results are shown in Figure 9. The results showed that there was a high correlation between ventral sulcus depth and slice area, and a weak positive correlation with grain width and thickness. Grain weight had a strong positive correlation with length, width, thickness, volume, and surface area. The wheat grain weight was measured by high precision electronic balance, and the weight of single wheat grain was approximately between 25 and 50 mg. Since the wheat grain weight was of great significance to the final yield, five different regression algorithms were applied in the study to predict grain weight with 500 grains in five wheat varieties, which included multivariable linear regression, Bayesian Ridge (BR), KNN regression, random forest (RF) regression, and gradient boosting regression (GBR). All regression models were performed on Sklearn Tool Kit, and the super parameters were determined by grid search and learning curve method. Then, 10-fold cross-validation method was used to evaluate each model, in which the dataset was randomly divided into 10 parts, while 9 of them were taken as the training set in turn, and the rest as the test set, and the average of the 10 results was computed as the model accuracy. The performance of grain weight prediction models is shown in Table 4, and the GBR model had the best performance, the R^2^, MAPE, and RMSE of which was 0.83 and 3.37%, and 2.45 mg, respectively. The original data of five grain weight prediction models using 500 wheat grains are shown in Supplementary Table 3.

**TABLE 4:** The performance of grain weight prediction models.

Regression method	*R* ^2^	MAPE (%)	RMSE (mg)
LR	0.7698	4.11	2.83
BR	0.7899	4.02	2.65
KNN	0.7956	3.81	2.65
RF	0.8103	3.68	2.45
GBR	0.8345	3.37	2.45

### Efficiency Evaluation

Since the optimal rotation angle of the turntable was 30° determined by the orthogonal experiment, each measurement that includes 25 wheat grains should acquire point clouds 12 times, while each acquisition would take about 14 s, and the entire acquisition would take about 3 min for each measurement. Then, it took about 2 min to apply the point cloud algorithms. Therefore, the measurement of 25 wheat grains took about 5 min in total, and the average efficiency was 12 s per grain. In the X-ray CT contrast experiment of wheat, it takes about 6 min to acquire 1,440 projection images of 25 grains and about 2 min to reconstruct the 3D point clouds. Similarly, the processing of point cloud algorithms costs about 2 min, so the total measurement takes about 10 min, and the average efficiency is 24 s per grain. The efficiency of manual measurement in the experiment was about 120 s per grain, but not including the complex grain traits. Therefore, the efficiency of structured light imaging system was two times of X-ray CT imaging system and 10 times of manual measurement, which demonstrated a non-destructive method with high efficiency.

## Discussion

With the rapid growth of the world population, wheat breeding and yield improvement are urgent to meet the growing demand. The wheat grain weight would determine the final yield, and the grain phenotypic traits that include grain length, width, thickness, volume, are closely related to grain weight. Meanwhile, the ventral sulcus, wheat unique morphological feature, is of great significance to the flour yield, which is hard to be measured by conventional methods. Since most wheat breeding research is generally based on the single grain, single grain phenotypic trait measurement that includes 3D shape and ventral sulcus is vital and necessary. At present, most grain phenotype detection methods were based on the two-dimensional images and provided phenotypic information about population seeds (Dubey et al., 2006; Anami et al., 2009). In this paper, an intelligent analysis method based on the structured light imaging has been proposed to extract the 3D wheat grain phenotypes and ventral sulcus traits.

To determine the best experimental conditions, three levels of orthogonal experiments were carried out on the three influencing factors that include background color, rotation angle, and scanning angle. As could be seen from Table 3, the R value of stage color factor was the largest. It showed that the background has the greatest influence on structured light scanning. For the factor of rotation angle, it can be seen that the difference between K_1_ and K_2_ was significantly greater than that between K_2_ and K_3_. It could be convinced that blindly increasing the rotation angle was limited to improve the accuracy; on the contrary, it would reduce the measurement efficiency. Similarly, for the scanning angle, the effect was basically the same within a certain range. Finally, it indicated that the structure light system in this study had the best performance in the condition of the black object stage, 30° rotation angle, and 37° scanning angle.

**FIGURE 10:**
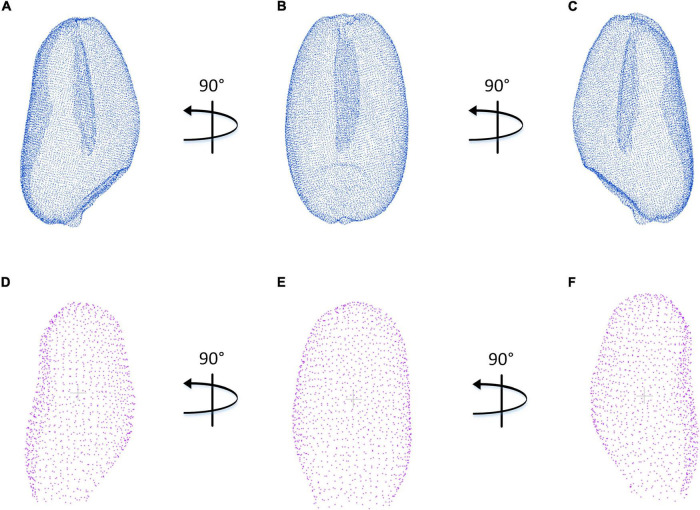
Comparison of wheat grain point clouds obtained by X-ray CT and structured light imaging. **(A–C)** X-ray CT system. **(D–F)** Structured light system.

The contrast experiment between structured light imaging and X-ray CT imaging was carried out. Figures 10A–F indicates three main directions of the side view in the wheat grain point cloud, which was generated by the PCA algorithm and coordinate transformation. Although the point cloud density reconstructed by X-ray CT system was significantly higher than that of structured light system, structured light imaging was able to achieve similar accuracy in the length, width, and thickness measurement, which could be demonstrated in Figures 7A–F. Moreover, compared with CT imaging, the structured light imaging had the advantages of higher efficiency, lower cost, and non-radiation. In this study, the structured light imagining system collected 25 wheat grain point clouds at a time and the data acquisition would takes about 3 min for each measurement. Then, it took about 2 min for data analysis. Therefore, the average efficiency of structured light imaging system was 12 s per grain which was about two times of X-ray CT imaging system. In fact, data acquisition and data analysis could be processed in parallel. If only the time spent on data acquisition was calculated, the efficiency was 4,000 grains per day based on the standard of working 8 h a day. It cost about $20,000 to build the structured light imagining system, which was about one-tenth of the CT system. Therefore, this method was demonstrated to be an efficient and cost-effective way for wheat breeding research.

Since the wheat grain weight was of great significance to the final yield, five different regression algorithms were applied in the study to predict grain weight with 500 grains in five wheat varieties, which include multivariable linear regression, BR, KNN regression, RF regression, and GBR. As shown in Table 4, *R*^2^ ranged from 0.77 to 0.83, and the results showed that the integrated regression algorithms that include RF and GBR would significantly promote the *R*^2^, compared with the other independent regression algorithms, and the *R*^2^ were greater than 0.8. The GBR had the best performance with *R*^2^ = 0.83, which had adopted the residual optimization iteration method to improve the wheat grain weight model.

## Conclusion

In this study, a novel method for wheat ventral sulcus measurement was proposed based on the structured light imaging. A total of 32 phenotypic characters of wheat grain were extracted by the specialized point cloud processing algorithms, and the prediction model of wheat grain weight was developed. The results showed that the structured light measurement of wheat grain traits, which include the 3D wheat grain phenotypes and ventral sulcus traits, had a high consistence with the manual measurement. Compared with X-ray CT and manual measurement, the efficiency of the method was significantly improved. Based on 32 wheat grain traits, the GBR model was selected to achieve the wheat grain weight prediction. Meanwhile, a specialized software was developed, in which wheat grain phenotype extraction algorithm and optimal grain weight prediction model were integrated. The software could automatically segment single wheat grain, obtain the ventral sulcus slice, and display the results visually. In conclusion, this study has provided a new software that could automatically achieve wheat grain 3D phenotypic traits extraction and grain weight prediction, which was convenient and useful for wheat researcher.

## Data Availability Statement

The datasets presented in this study can be found in online repositories. The names of the repository/repositories and accession number(s) can be found in the article/Supplementary Material.

## Author Contributions

CH and ZQ designed the research, performed the experiments, analyzed the data, and wrote the manuscript. ZZ and XH performed the experiments. WX, XL, and PS revised the manuscript. WY supervised the project and wrote the manuscript. All authors contributed to the article and approved the submitted version.

## Conflict of Interest

The authors declare that the research was conducted in the absence of any commercial or financial relationships that could be construed as a potential conflict of interest.

## Publisher’s Note

All claims expressed in this article are solely those of the authors and do not necessarily represent those of their affiliated organizations, or those of the publisher, the editors and the reviewers. Any product that may be evaluated in this article, or claim that may be made by its manufacturer, is not guaranteed or endorsed by the publisher.

## Footnotes

1
https://pan.baidu.com/s/1ujRy92FgSPfqsZSsFyApxQ

## Supplementary Material

Supplementary Table 1 The original data of the orthogonal experiment data of 125 wheat grains.

Supplementary Table 2 The original data of structured light imaging, X-ray CT, and manual measurements of 125 wheat grains.

Supplementary Table 3 The original data of five grain weight prediction models using 500 wheat grains.

Supplementary Video 1 Introduce the use of the wheat 3D traits extraction software.

Supplementary Appendix 1 Parameters of X-ray source and detector.

Supplementary Appendix 2 Comparison of downsampling accuracy (Luyuan 118).
