# Supplementary material for: An Intelligent Analysis Method for 3D Wheat Grain and Ventral Sulcus Traits Based on Structured Light Imaging
Source: Front Plant Sci. 2022 Apr 13;13:840908. doi: 10.3389/fpls.2022.840908 (PMC9044079; doi:10.3389/fpls.2022.840908)
Supplement: Supplementary Appendix 1 — Parameters of X-ray source and detector. [file Data_Sheet_1.docx]

**Supplementary Tables**

Table A1. Parameters of X-ray source and detector

| Parameter | Value |
| --- | --- |
| Tube voltage | 60kV |
| Tube current | 300$\mu$A |
| X-ray angel | 22.5° |
| Distance between X-ray tube and detector | 1181.010mm |
| Distance between X-ray tube and rotating table | 198.718mm |
| Image magnification | 5.94 |
| Image resolution | $1430\times1430$pixels |
| Pixel size | 150$\mu$m |
| A/D conversion | 16bit |

Table A2. Comparison of down sampling accuracy (Luyuan 118)

|  | | Down sampling | | | | No Down sampling | | | | Ground Truth | | | |
| --- | --- | --- | --- | --- | --- | --- | --- | --- | --- | --- | --- | --- | --- |
| No. | Length  (mm) | | | Width  (mm) | Thickness  (mm) | Length  (mm) | Width  (mm) | Thickness  (mm) | Length  (mm) | | Width  (mm) | Thickness  (mm) |  |
| 1 | 6.4163 | | | 3.6021 | 3.2478 | 6.4312 | 3.6164 | 3.2724 | 6.7 | | 3.68 | 3.16 |  |
| 2 | 7.2043 | | | 3.7283 | 3.3129 | 7.2488 | 3.7497 | 3.3426 | 7.37 | | 3.84 | 3.2 |  |
| 3 | 7.0858 | | | 3.6395 | 3.1901 | 7.1406 | 3.6712 | 3.2108 | 7.03 | | 3.62 | 3.11 |  |
| 4 | 7.5717 | | | 3.6233 | 3.4144 | 7.5719 | 3.6298 | 3.4251 | 7.58 | | 3.63 | 3.38 |  |
| 5 | 6.9372 | | | 3.7127 | 3.4241 | 6.9715 | 3.7191 | 3.4351 | 7.09 | | 3.74 | 3.34 |  |
| 6 | 6.4835 | | | 3.7571 | 3.5197 | 6.53 | 3.7761 | 3.5223 | 6.57 | | 3.82 | 3.44 |  |
| 7 | 6.7958 | | | 3.5389 | 3.2079 | 6.8231 | 3.5678 | 3.227 | 6.85 | | 3.61 | 3.13 |  |
| 8 | 7.3511 | | | 3.6176 | 3.2190 | 7.3728 | 3.6186 | 3.2352 | 7.34 | | 3.67 | 3.18 |  |
| 9 | 7.5658 | | | 3.7430 | 3.4784 | 7.5825 | 3.7616 | 3.4955 | 7.37 | | 3.69 | 3.34 |  |
| 10 | 7.0114 | | | 3.4742 | 3.0739 | 7.0576 | 3.4863 | 3.0846 | 6.87 | | 3.53 | 3 |  |
| 11 | 7.3806 | | | 3.6686 | 3.4440 | 7.3878 | 3.6756 | 3.4654 | 7.54 | | 3.7 | 3.42 |  |
| 12 | 6.9551 | | | 3.5631 | 3.1736 | 6.9777 | 3.5752 | 3.1954 | 6.98 | | 3.62 | 3.13 |  |
| 13 | 6.9380 | | | 3.6470 | 3.2138 | 6.9627 | 3.6537 | 3.2287 | 7.05 | | 3.68 | 3.16 |  |
| 14 | 7.2265 | | | 3.7795 | 3.3775 | 7.2622 | 3.8076 | 3.4072 | 7.31 | | 3.9 | 3.26 |  |
| 15 | 7.2898 | | | 3.7562 | 3.3431 | 7.3218 | 3.7597 | 3.3442 | 7.07 | | 3.67 | 3.27 |  |
| 16 | 7.2869 | | | 3.6235 | 3.3884 | 7.3411 | 3.6535 | 3.3917 | 7 | | 3.69 | 3.24 |  |
| 17 | 7.0867 | | | 3.5795 | 3.3184 | 7.1055 | 3.5995 | 3.3245 | 7.11 | | 3.67 | 3.24 |  |
| 18 | 7.5921 | | | 3.6932 | 3.2356 | 7.6205 | 3.7177 | 3.237 | 7.44 | | 3.69 | 3.21 |  |
| 19 | 6.6154 | | | 3.6091 | 3.1891 | 6.6286 | 3.6129 | 3.198 | 6.62 | | 3.56 | 3.08 |  |
| 20 | 6.9297 | | | 3.6856 | 3.3980 | 6.9735 | 3.7079 | 3.412 | 6.98 | | 3.56 | 3.27 |  |
| 21 | 7.5001 | | | 3.7479 | 3.4194 | 7.5015 | 3.7799 | 3.4235 | 7.3 | | 3.84 | 3.38 |  |
| 22 | 6.7812 | | | 3.8965 | 3.2026 | 6.8031 | 3.9347 | 3.2272 | 6.7 | | 3.87 | 3.14 |  |
| 23 | 7.4270 | | | 3.6295 | 3.3860 | 7.4451 | 3.6435 | 3.3907 | 7.4 | | 3.64 | 3.31 |  |
| 24 | 7.3342 | | | 3.5826 | 3.1895 | 7.3809 | 3.5877 | 3.218 | 7.48 | | 3.54 | 3.06 |  |
| 25 | 6.5712 | | | 3.4486 | 3.2721 | 6.5832 | 3.4669 | 3.3022 | 6.87 | | 3.53 | 3.21 |  |
| MAPE | | | 1.71% | 1.58% | 2.46% | 1.66% | 1.45% | 2.93% |  | |  |  |  |
